# Supplementary material for: Chromothripsis during telomere crisis is independent of NHEJ, and consistent with a replicative origin
Source: Genome Res. 2019 May;29(5):737–49. doi: 10.1101/gr.240705.118 (PMC6499312; doi:10.1101/gr.240705.118)
Supplement: Supplemental Material [file supp_gr.240705.118_Supplemental_file_1.zip › contigs/annotated_contigs/DB73/contig.2.DB73_length_521_mean_cov_6.50863723608.docx]

**DB73_length_521_mean_cov_6.50863723608**

```
CCTCATGGCACAGTGAACTTTTGGTTTAACTACTGGAATGTTCAGTCAAAACCAACATGTTGCACACTCAGCTTTTTTTTTTTTGAGAC
 >chr5:41353123-41353329 + E=1e-102
AGAGTCTCGCTCTGTTGCCCAGGCTGGAGTGCAGTGGCGCGATCTCCGCTCACTGCAAGCTCCACCTCCCGGGTTCATGCCATTCTCCT

GACTCAGCCTCCCGAGTAGCTG|G|AAAATCTGTCACAGTTGACTTTAATTAGTTGGTCTGTGGGGGCTCAGTTGCTCAAAGGAAGAAA
                       >chr5:41333088-41333409 - E=1e-181
GATCCATGATCATTATTTTAAACTTTGCAGATCAATGAAGCCTTCATTTCTTAGGCTGGGTTGGGACGGGCAGTCACAAGCCATTGACG

TTTTTGTTTGTTTGTTTGTTTGTTTGTTTTTGTCTTTTATTTTATATTATTTTATTATTATTATACTTTAAGTTCTAGGGTACATGTGC

ACAATGTGCAGGTTAGTTACAAATGTATACATGTGCCATGCTGGTGTGCTGCACCCGTTAACTCGTCATTTAGCATTA
```
